# Hereditary cold-induced palmar erythema with dysesthesia: A novel entity with response to onabotulinumtoxinA injections

**DOI:** 10.1016/j.jdcr.2024.06.017

**Published:** 2024-07-06

**Authors:** Injae Jung, Shena Kravitz, John Linabury

**Affiliations:** a School of Medicine, Uniformed Services University, Bethesda, Maryland; b Department of Dermatology, Walter Reed National Military Medical Center, Bethesda, Maryland

**Keywords:** autoimmune, erythema palmare hereditarium, erythromelalgia, onabotulinumtoxinA, palmar erythema, Raynaud's phenomenon

## Introduction

Erythema palmare hereditarium (EPH) is a rare, heritable subtype of palmar erythema (PE) that usually presents as a symmetric, nonpainful, nonpruritic erythema affecting the bilateral palms. EPH typically appears at birth or early childhood but can also appear later in life.^1^ To the best of our knowledge, this is one of the first reported cases of suspected EPH with Raynaud’s phenomenon like cold-induced PE and dysesthesia responsive to onabotulinumtoxinA injections.^2^

## Case report

**Fig 1:**
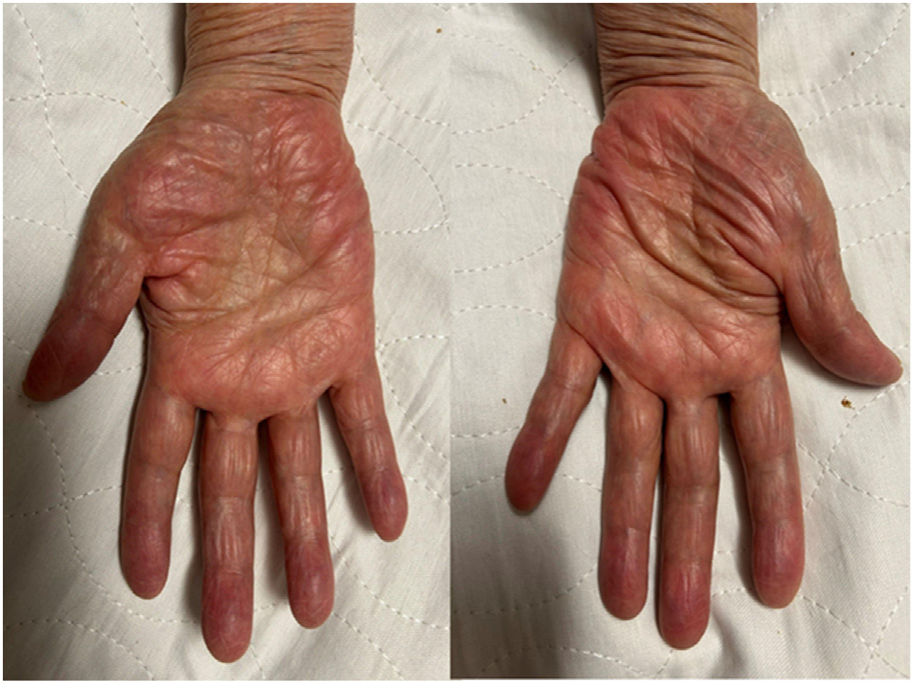
Palmar erythema of bilateral hypothenar eminences in the patient's mother.

A 56-year-old female with a history of cervical disc disease and essential tremors presented to the dermatology clinic with a 15-year history of intermittent erythematous patches on bilateral hypothenar eminences. She endorsed a burning sensation and erythema of the bilateral hypothenar eminences when the affected areas were exposed to cold surfaces. Prior to the onset of symptoms, there was no pallor or cyanotic skin changes as is often observed in Raynaud’s phenomenon. All digits were spared. Symptoms were relieved by wearing gloves or warming her hands. The patient wore gloves while working on her computer due to these symptoms. Her medications included propranolol, celecoxib, hyoscyamine, primidone, and sertraline. Of note, her symptoms began before starting propranolol for essential tremors and did not worsen after starting this medication. Additionally, the patient reported similar symptoms of cold-induced dysesthesia and PE in her mother that started in her 50s (Fig 1).

**Fig 2:**
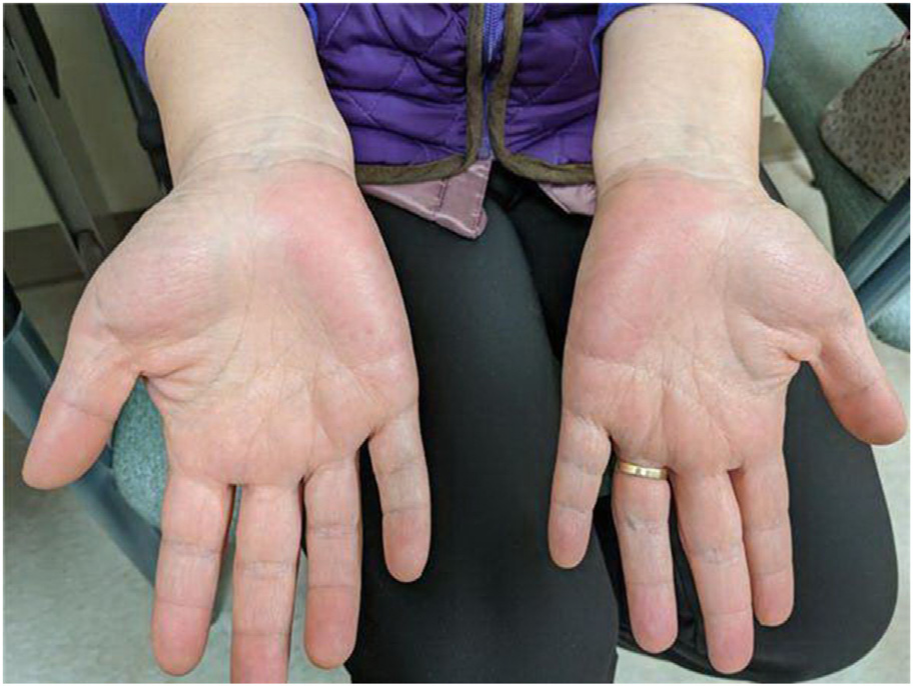
Palmar erythema of bilateral hypothenar eminences in patient at initial presentation.

**Fig 3:**
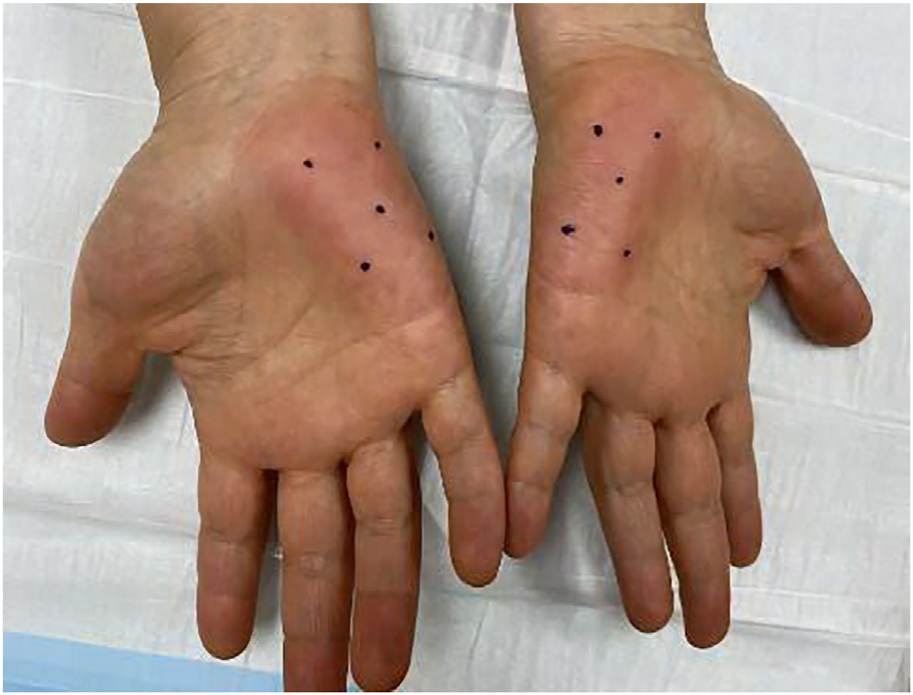
Palmar erythema being managed with a trial of onabotulinumtoxinA injections.

Physical exam was notable for erythematous patches without epidermal changes along the bilateral hypothenar eminences (Fig 2). The patient’s cryoglobulin serum and autoimmune hemolytic anemia workup was unremarkable. Antinuclear antibody, rheumatoid factor, human immunodeficiency virus, hepatitis screen, complement levels, and nerve conduction testing were unremarkable. The review of systems was unremarkable and her age-appropriate cancer screen was up to date. Skin punch biopsy of the affected area showed normal acral skin without leukocytoclastic vasculitis. While a skin biopsy of EPH usually shows dilated vessels throughout the dermis,^3^ a clinical diagnosis of EPH was made in the setting of her permanent bilateral PE, family history of similar findings, and negative underlying workup. However, this patient also had unique symptoms of Raynaud’s phenomenon-like cold-induced dysesthesia. Since the patient’s cold-induced dysesthesia symptoms were impacting patient’s quality of life despite conservative measures, the patient was treated with off-label onabotulinumtoxinA injections at 2.5 U/0.1 mL per injection with 5 injection sites in each hypothenar eminence (Fig 3). The patient reported near complete improvement of her symptoms lasting approximately 6 months without side effects, including hand weakness. This greatly improved her quality of life as she no longer needed to wear gloves while working on her computer. After 9 months status-post treatment, the patient reported only 10% to 15% of her symptoms returning.

## Discussion

EPH was first named in 1929 by John E. Lane to describe an asymptomatic, bright-red coloration of bilateral thenar and hypothenar eminences with an inheritable pattern.^4^ EPH is typically a diagnosis of exclusion. Ruling out underlying infections, autoimmune disease, malignancy, and culprit medications is imperative in the workup for PE as there are many secondary causes.^5^ Hepatic decompensation resulting in hyperestrogenism is known to increase vascularity that leads to PE.^5^ Autoimmune disorders associated with PE include rheumatoid arthritis, systemic lupus erythematosus, sarcoidosis, and autoimmune hemolytic anemia. Endocrinopathies such as diabetes mellitus or hypothyroidism are also implicated. PE seldom presents as paraneoplastic finding associated with brain, breast, colon, pancreas, and hepatic neoplasms likely due to tumor angiogenesis.^5^ PE has been reported in infected individuals with human T-lymphocytic virus-1 myelopathy, brucellosis, trichinellosis, and newborns with gestational syphilis.^5^ Most common medications believed to cause PE include amiodarone, statins, and beta-blockers.^5^

In the setting of her negative underlying workup and maternal history of similar findings, the patient’s symptoms are most consistent with a form of EPH with overlapping features of erythromelalgia and Raynaud’s phenomenon. However, her symptoms are unique in that they did not begin until her 40s (with similar time of onset for the patient’s mother). The patient’s dysesthesia is similar to erythromelalgia, but instead of erythema and burning induced by heat and alleviated by cooling,^6^ this patient’s symptoms were exacerbated by cold. While Raynaud’s phenomenon remained on the differential diagnosis, the patient denied the usual ischemic, hypoxic, hyperemic color changes typically seen in Raynaud’s phenomenon.^7^ Furthermore, the hypothenar eminences are not commonly affected sites in Raynaud’s phenomenon. As far as the authors are aware, this is the first reported case of a form of hereditary, cold-induced PE, and dysesthesia with overlapping features of Raynaud’s phenomenon.

OnabotulinumtoxinA has been used to treat a variety of muscular and vascular spastic disorders through its ability to block smooth muscle vascoconstriction.^8^ Recent case reports have also shown promise in mitigating erythromelalgia-related pain with onabotulinumtoxinA injections.^9^ The presumed mechanism is inhibition of pain-relaying neurotransmitters from peripheral nerve endings, as onabotulinumtoxinA injections are also used for symptomatic relief in those with trigeminal neuropathy and chronic regional pain syndrome.^9^ This patient’s significant improvement in symptoms after onabotulinumtoxinA injections further suggests that this patient’s symptoms are driven by inappropriate vasospasm in response to cold temperatures. The authors propose the term “hereditary cold-induced erythromelalgia'” for this new entity. Furthermore, this case highlights onabotulinumtoxinA injections as an effective and long-lasting treatment for this entity.

## Conflicts of interest

None disclosed.
